# Supplementary material for: Real-Time Genomic Surveillance during the 2021 Re-Emergence of the Yellow Fever Virus in Rio Grande do Sul State, Brazil
Source: Viruses. 2021 Oct 1;13(10):1976. doi: 10.3390/v13101976 (PMC8539658; doi:10.3390/v13101976)
Supplement: Supplementary file 1 [file viruses-13-01976-s001.zip › viruses-1386859-supplementary 2.pdf]

viruses

MDPI

Supplementary Materials

Real-Time Genomic Surveillance during the 2021 Re-Emergence of the Yellow Fever Virus in Rio Grande do Sul State and Brazil

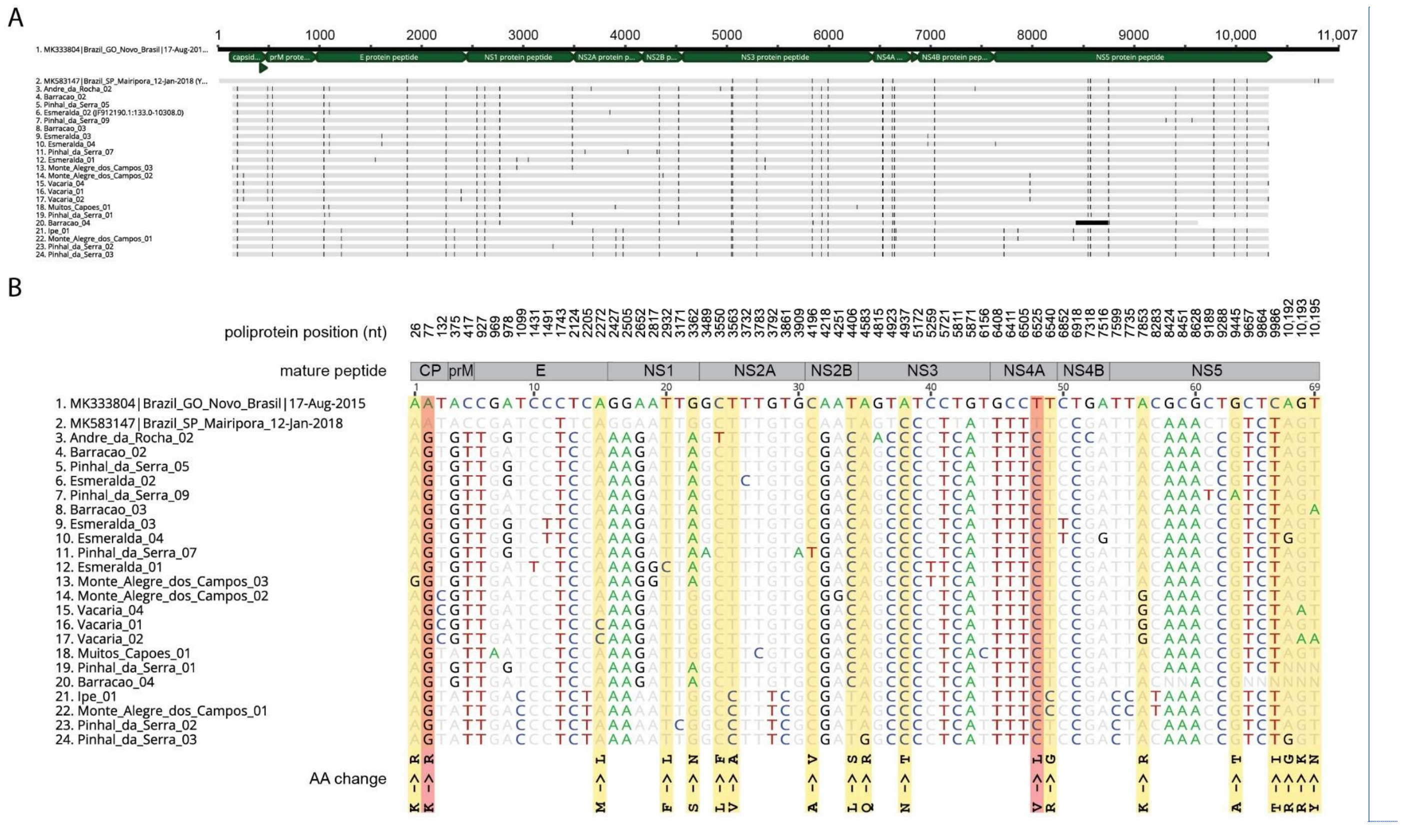

Figure S1. Sequence alignment of the yellow fever virus genomes detected in this study compared to a genome reference.
